# Supplementary material for: Parental Microbiota Modulates Offspring Development, Body Mass and Fecundity in a Polyphagous Fruit Fly
Source: Microorganisms. 2020 Aug 24;8(9):1289. doi: 10.3390/microorganisms8091289 (PMC7563405; doi:10.3390/microorganisms8091289)
Supplement: Supplementary file 1 [file microorganisms-08-01289-s001.pdf]

Supplementary document for article:

Parental microbiota modulates offspring development, body mass and fecundity in a polyphagous fruit fly

Binh Nguyen[1], Anh Than[1,2], Hue Dinh[1], Juliano Morimoto[1,3], Fleur Ponton[1*]

[1] Department of Biological Sciences, Macquarie University, Sydney, NSW, Australia
[2] Department of Entomology, Vietnam National University of Agriculture, Trau Quy, Gia Lam Hanoi, Vietnam
[3] School of Biological Sciences, Zoology Building, Tillydrone Ave, Aberdeen AB24 2TZ, United Kingdom
*Corespondence: fleur.ponton@mq.edu.au

Received: date; Accepted: date; Published: 24 August 2020

Supplementary information 1

Culture-dependent method and PCR quantification to assess microbial status of parental axenic and control samples at egg, larval, and adult stages

Samples were collected after egg washing, at third instar larvae, and at one-day-old adult. Eggs were pooled in groups of ca. 1500 eggs (100µL, N = 3) while larvae were collected in groups of 20 (N = 3) and adults were assessed individually (N = 3 males, 3 females). Larvae and adults were freeze-killed for 2h before subjecting to the surface sterilization by one wash for 3 min in 0.5% bleach (Peerless JAL®), followed by one wash in 70% ethanol for 1 min and three washes in sterile miliQ water. Egg, larval, and adult samples were then homogenated in 0.4, 0.1, and 1mL sterile PBS buffer, respectively and 25µL of each homogenate was plated on a petri dish contains 25 mL of either Luria Bertani agar (cat. no., 22700-025, Life technologies), or Man-Rogosa-Sharpe agar (cat. no., CM0361, Oxoid®), or Potato-Dextrose agar (cat. no., CM0139, Oxoid®) using single use L-shape spreaders (cat. no., Z723193, Sigma). LB dishes were incubated at 28˚C for 24-48h, MRS and PDA dishes at 28˚C for 48-72h. The number of colony-forming unit (CFU) on LB, MRS, PDA dishes was measured. The total CFU number per sample was then averaged between the 3 replicates for each medium (see Table S3).

The remained homogenates were subjected to PCR to quantified the amount of 16S rRNA gene (represents for the presence of bacteria) and the internal transcribed spacer (ITS, represents for the presence of fungi) with washing chemicals (bleach, ethanol, and sterile water, N = 3 replicates, N *total* = 9) used as negative control. The initial PCR amplicons were generated using AmpliTaq Gold 360 mastermix (Life Technologies, Australia). PCR conditions and primers were outlined in Table S2 below. A secondary PCR to index the amplicons was performed with TaKaRa Taq DNA Polymerase (Clontech). The resulting amplicons were measured by florometry (Invitrogen Picogreen) and normalised. The eqimolar pool was then measured by qPCR (KAPA). 16S rRNA and ITS concentrations (ng/µL) were then averaged between the 3 replicates (see Table S4).

Supplementary figures

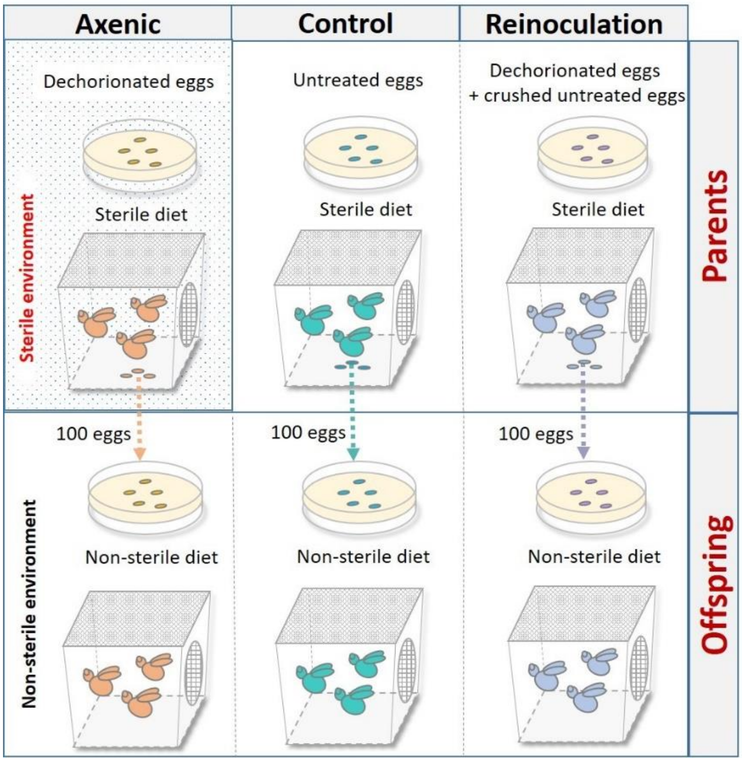

Figure S1 – Schematic representation of the experimental design

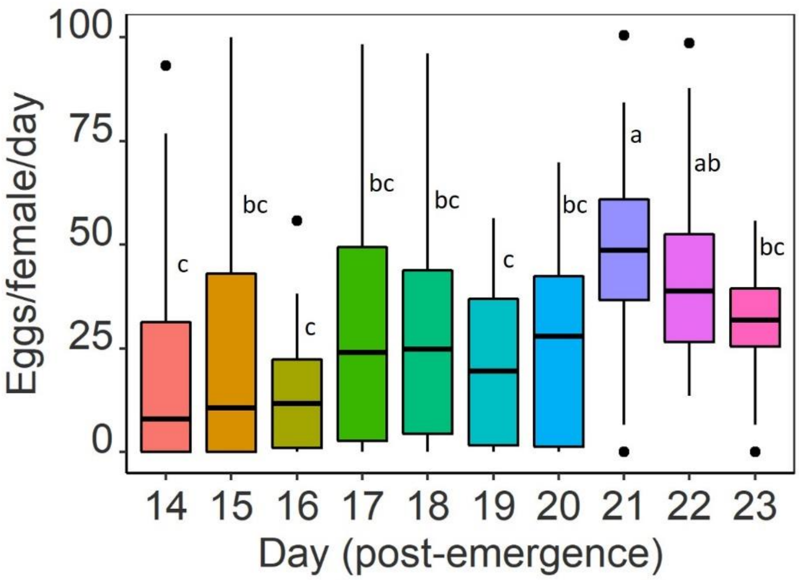

Figure S2. Effects of time on egg production in offspring. Different letters indicate a significant difference between the treatments (SNK *post hoc* test, p = 0.05)

Supplementary tables

Table S1. Diet recipe (for 1000 mL) and suppliers [2]

**Ingredient**	**Amount**	**Supplier**
Brewer's yeast (g)	204	SF Health foods, Australia
Sugar (g)	121.8	Homebrand, Australia
Agar (g)	10	MP Biomedicals
Citric Acid (g)	23	Sigma, Australia
Nipagin (g)	2	Southern Bio- logical, Australia
Sodium Benzoate (g)	2	Sigma, Australia
Wheat Germ Oil (mL)	2	Melrose laboratories PTY LTD, Australia
MiliQ water (mL)	1000	Lab source

Table S2. PCR conditions and primer sequences

**PCR conditions**						
Target	Cycle	Initial	Disassociate	Anneal	Extension	Finish
16S: V1- V3	29	95˚C for 7 min	94˚C for 45s	50˚C for 60S	72˚C for 60S	72˚C for 7 min
ITS1F – ITS2	35	95˚C for 7 min	94˚C for 30s	55˚C for 45S	72˚C for 60S	72˚C for 7 min

**Primer sequences**		
Target	16S: V1- V3	ITS1F – ITS2
Forward	AGAGTTTGATCMTGGCTCAG	CTTGGTCATTTAGAGGAAGTAA
Reverse	GWATTACCGCGGCKGCTG	GCTGCGTTCTTCATCGATGC

Table S3. Total CFU count per sample of parental axenic and control treatments was averaged between 3 replicates for each culture medium.

Treatment	Stage	Sex	MRS (average± SE)	LB (average± SE)	PDA (average± SE)
Parental control	Egg (n=1500)	-	687 (241)	3202 (450)	0 (0)
Parental axenic	Egg (n=1500)	-	0 (0)	0 (0)	0 (0)
Parental control	Larval (n=20)	-	11 (3)	16 (10)	8 (8)
Parental axenic	Larval (n=20)	-	0 (0)	0 (0)	0 (0)
Parental control	Adult (n=1)	Male	347 (307)	133 (133)	0 (0)
Parental control	Adult (n=1)	Female	960 (257)	1472 (658)	93 (93)
Parental axenic	Adult (n=1)	Male	0 (0)	0 (0)	0 (0)
Parental axenic	Adult (n=1)	Female	0 (0)	0 (0)	0 (0)

Table S4. 16S rRNA and ITS concentration (ng/µL) for 3 replicates of parental axenic and control treatments and washing chemicals.

Treatment	Stage	Sex	16S rRNA (average± SE)	ITS (average± SE)
Parental control	Egg (n=1500)	-	8.56 (1.89)	0 (0)
Parental axenic	Egg (n=1500)	-	0 (0)	0 (0)
Parental control	Larval (n=20)	-	0.12 (0.06)	0.08 (0.08)
Parental axenic	Larval (n=20)	-	0 (0)	0 (0)
Parental control	Adult (n=1)	Male	1.22 (0.43)	0 (0)
Parental control	Adult (n=1)	Female	2.92 (1.65)	0.07 (0.07)
Parental axenic	Adult (n=1)	Male	0 (0)	0 (0)
Parental axenic	Adult (n=1)	Female	0 (0)	0 (0)
0.5% bleach	-	-	0 (0)	0 (0)
70% ethanol	-	-	0 (0)	0 (0)
MiliQ water	-	-	0 (0)	0 (0)

Table S5. Mean (± SE) of egg hatching success and developmental time of parental flies

Treatment	n	Egg hatching (%)	Developmental time (day)
Parental axenic	10	90.4 (0.37)	17.44 (0.04)
Parental control	10	89.7 (0.97)	17.41 (0.05)
Parental reinoculation	10	88.5 (0.9)	17.44 (0.04)

References

1. Koyle ML, Veloz M, Judd AM, Wong AC-N, Newell PD, Douglas AE, Chaston JM. 2016 Rearing the Fruit Fly Drosophila melanogaster Under Axenic and Gnotobiotic Conditions. *J. Vis. Exp.* , 1–8. (doi:10.3791/54219)

2. Moadeli T, Taylor PW, Ponton F. 2017 High productivity gel diets for rearing of Queensland fruit fly, Bactrocera tryoni. *J. Pest Sci. (2004).* **90**, 507–520. (doi:10.1007/s10340-016-0813-0)

© 2019 by the authors. Licensee MDPI, Basel, Switzerland. This article is an open access article distributed under the terms and conditions of the Creative Commons Attribution (CC BY) license (http://creativecommons.org/licenses/by/4.0/).
